# Identification of novel protein biomarkers from the blood and urine for the early diagnosis of bladder cancer via proximity extension analysis

**DOI:** 10.1186/s12967-024-04951-z

**Published:** 2024-03-26

**Authors:** Tong Kong, Yang Qu, Taowa Zhao, Zitong Niu, Xiuyi Lv, Yiting Wang, Qiaojiao Ding, Pengyao Wei, Jun Fu, Liang Wang, Jing Gao, Cheng Zhou, Suying Wang, Junhui Jiang, Jianping Zheng, Kaizhe Wang, Kerong Wu

**Affiliations:** 1 Ningbo Cixi Institute of BioMedical Engineering, Ningbo Institute of Materials Technology & Engineering, Chinese Academy of Sciences (CAS), Ningbo, 315300 People’s Republic of China; 2 Department of Urology, Key Laboratory of Translational Research for Urology of Ningbo City, Key Laboratory of Precision Medicine for Atherosclerotic Diseases of Zhejiang Province, The First Affiliated Hospital of Ningbo University (Ningbo First Hospital), Ningbo, Zhejiang China; 3 LC-Bio Technology Co., Ltd., Hangzhou, China; 4 Olink Proteomics, Shanghai, China; 5 Ningbo Clinical Pathology Diagnostic Centre, Ningbo, Zhejiang China

**Keywords:** Bladder cancer, Diagnostic model, Biomarkers, Diagnosis, Prognosis proximity extension assay (PEA)

## Abstract

**Background:**

Bladder cancer (BC) is a very common urinary tract malignancy that has a high incidence and lethality. In this study, we identified BC biomarkers and described a new noninvasive detection method using serum and urine samples for the early detection of BC.

**Methods:**

Serum and urine samples were retrospectively collected from patients with BC (n = 99) and healthy controls (HC) (n = 50), and the expression levels of 92 inflammation-related proteins were examined via the proximity extension analysis (PEA) technique. Differential protein expression was then evaluated by univariate analysis (*p* < 0.05). The expression of the selected potential marker was further verified in BC and adjacent tissues by immunohistochemistry (IHC) and single-cell sequencing. A model was constructed to differentiate BC from HC by LASSO regression and compared to the detection capability of FISH.

**Results:**

The univariate analysis revealed significant differences in the expression levels of 40 proteins in the serum (p < 0.05) and 17 proteins in the urine (p < 0.05) between BC patients and HC. Six proteins (AREG, RET, WFDC2, FGFBP1, ESM-1, and PVRL4) were selected as potential BC biomarkers, and their expression was evaluated at the protein and transcriptome levels by IHC and single-cell sequencing, respectively. A diagnostic model (a signature) consisting of 14 protein markers (11 in serum and three in urine) was also established using LASSO regression to distinguish between BC patients and HC (area under the curve = 0.91, PPV = 0.91, sensitivity = 0.87, and specificity = 0.82). Our model showed better diagnostic efficacy than FISH, especially for early-stage, small, and low-grade BC.

**Conclusion:**

Using the PEA method, we identified a panel of potential protein markers in the serum and urine of BC patients. These proteins are associated with the development of BC. A total of 14 of these proteins can be used to detect early-stage, small, low-grade BC. Thus, these markers are promising for clinical translation to improve the prognosis of BC patients.

**Graphical Abstract:**

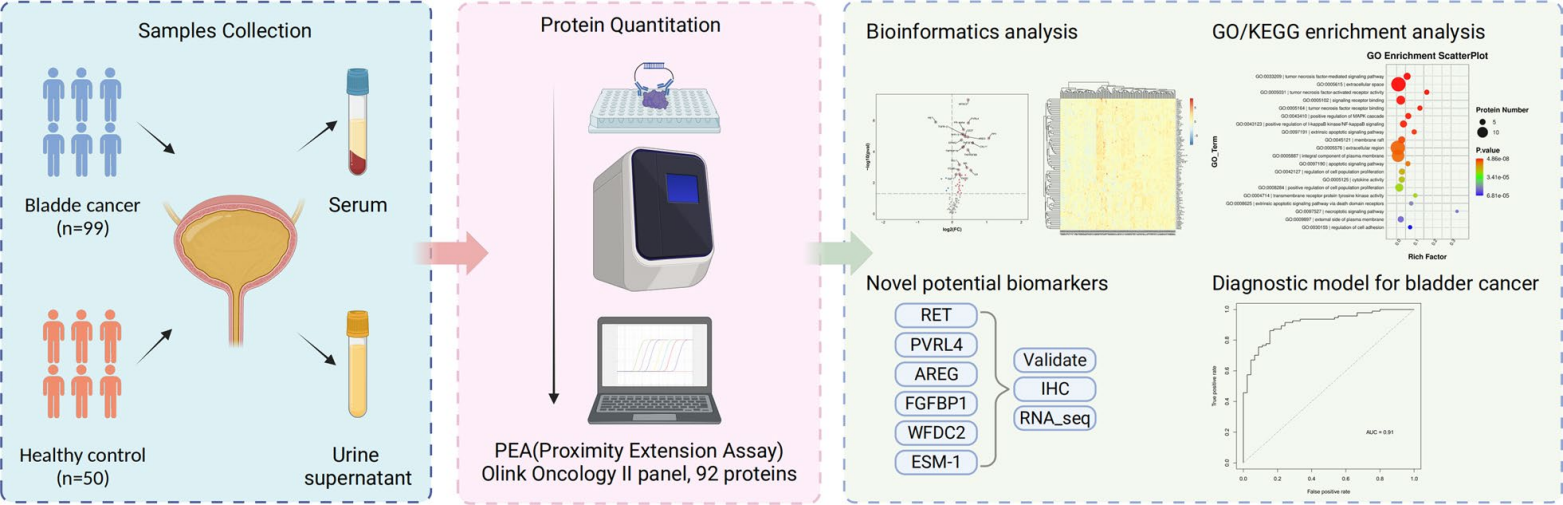

**Supplementary Information:**

The online version contains supplementary material available at 10.1186/s12967-024-04951-z.

## Background

Bladder cancer (BC) is one of the top 10 malignant tumors in the world and has a high incidence and mortality [1]. In 2020, approximately 573,000 new cases of BC were reported worldwide, and more than 212,000 people died from BC [2]. According to reports from the National Cancer Center of China (2022), there are approximately 82,300 new cases of BC and 33,700 deaths each year in China [3]. This disease can present as non-muscle-invasive bladder cancer (NMIBC), muscle-invasive bladder cancer (MIBC), or a metastatic form [4, 5]. Compared with NMIBC, MIBC often requires more complex and risky operations, involves more postoperative complications, and significantly decreases patient quality of life [1]. Therefore, researchers investigating BC have focused on its early screening and diagnosis.

The gold standard for diagnosing BC is cystoscopy and biopsy [4]. Cystoscopy is a highly sensitive but invasive and expensive procedure, and patients commonly complain of discomfort during the examination [6]. Urine cytology is a noninvasive and suitable procedure for accessing the eluent from an organ. However, it has low sensitivity for low-grade BC [7]. Fluorescence in situ hybridization (FISH) is a commonly used clinical examination technique with a sensitivity of 60–80%, but it also has low sensitivity for low-grade and/or small tumors [8]. Therefore, effective methods need to be developed for the early diagnosis of BC, especially for universal screening.

Liquid biopsy is a novel test for diagnosing diseases that analyses DNA, RNA, proteins, and other molecules in different biomarkers, such as plasma, urine, cerebrospinal fluid, and other fluids [9]. It is a noninvasive, highly sensitive, and flexible technique [10]. The detection of tumor-associated protein biomarkers is the most commonly used noninvasive method for the early detection of BC. It is effective in the screening, diagnosis, monitoring, and prognosis of BC, and the methods involve the use of nuclear matrix protein 22 (NMP22) [11], bladder tumor antigen-associated antigen (BTA stat and BTA trak) [12], AdxBladder [13], and Oncuria [14] Various methods have been developed that can screen proteins on a large scale. Proximity extension analysis (PEA) is a method that uses a qPCR readout and is used to analyze protein levels in plasma in longitudinal studies and genomic association studies. Many proteins with high selectivity and sensitivity are simultaneously measured by using minimal sample volumes (only 1 µL) and simple sample pretreatment.

In this study, we applied multiple PEA techniques to measure specific proteins in the serum and urine supernatant of BC patients. We analyzed the data via differential analysis. Machine learning was used to select different protein markers for constructing a BC diagnostic model. In this study, we identified a set of potential protein biomarkers and established a diagnostic model for BC, especially for the early screening of BC for clinical translation.

## Methods

### Study design and study patients

**Fig. 1:**
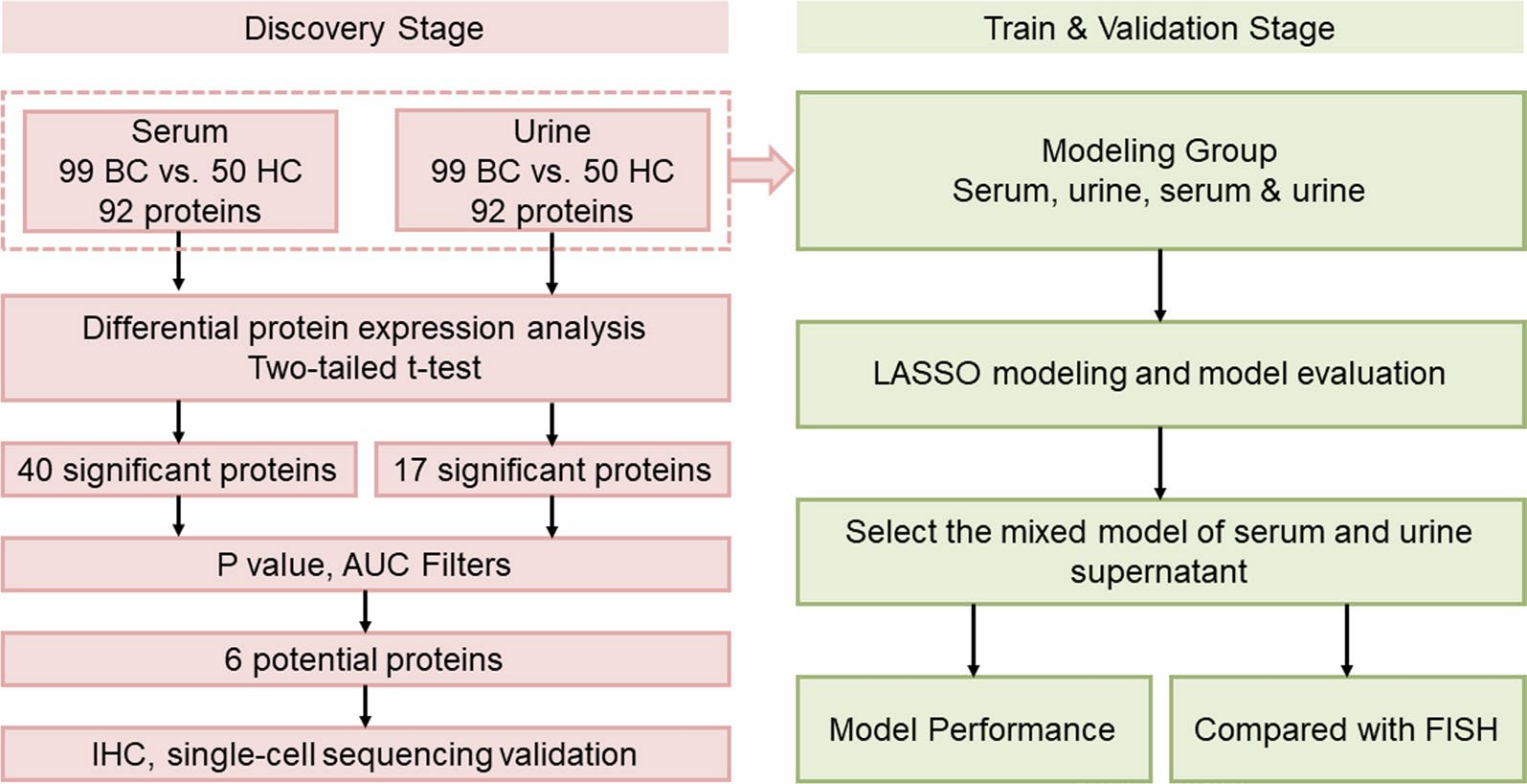
Strategies for serum and urinary supernatant Olink proteomics studies. Serum and urine supernatants were collected from BC patients (n = 99) and HC controls (n = 50) for the Olink-Oncology II panel. Bioinformatics analysis was subsequently performed to identify and validate potential bladder cancer liquid biopsy biomarkers. Early diagnosis of bladder cancer was achieved by the LASSO method, and model evaluation was performed. *B* bladder cancer, *HC* healthy control; *AUC* area under the curve

This study was approved by the ethics committee of The First Affiliated Hospital of Ningbo University, and informed consent was obtained from each patient before participation. In total, 99 patients who underwent surgery and pathology-verified BC were recruited at the Department of Urology in The First Affiliated Hospital of Ningbo University between July 2021 and August 2022. Serum and urine samples were collected after the patients were admitted to the hospital before surgery. Serum samples were aliquoted immediately after centrifugation and stored at − 80 °C until further use. The urine samples were centrifuged and stored at − 80 °C until further tests were conducted. The tumor stage and grade were reclassified following the 2017 TNM classification system and the 2004/2016 WHO grading system. We also included 50 healthy volunteers from the same hospital as controls in this study. Serum and urine samples were collected and processed as described above. Our research strategy for serum and urine supernatant samples from BC and HC subjects is depicted in Fig. 1.

### Protein analysis

The Olink^®^ OncologyII 96 × 96 panel (Olink Proteomics AB, Uppsala, Sweden) was used to simultaneously detect the expression levels of 92 proteins in the serum and urine supernatants of BC patients and healthy control participants. The PEA technique relies on a double recognition immunoassay. Two mutually matched antibodies labeled with unique DNA oligonucleotides bind simultaneously to target proteins in solution, hybridizing their DNA oligonucleotides, which serve as templates for the DNA polymerase-dependent extension step [15]. Complementary hybridization is required to generate the signal. The amplification and quantification of DNA were performed by microfluidic qPCR on a Fluidigm Biomark instrument. After the data were analyzed for quality control, they were normalized using internal extension controls and interplate controls to adjust for intra-assay and interassay variations. The final assay results are expressed as log2 values converted to protein expression (NPX) values; higher NPX values correspond to higher protein expression [16]. The signal specificity of this technique is very high, as complementary binding of two oligonucleotides is required to generate the signal. All validation data (detection limits, intra-assay precision, interassay precision, etc.) are available on the manufacturer’s website (www.olink.com). All the proteins included in the Oncology II assay are listed.

### *Fluorescence *in situ* hybridization and immunohistochemistry (IHC)*

The FISH technique uses fluorescence-labeled DNA probes for evaluating genetic changes in cells. Pathology samples can be examined because chromosomal aberrations associated with BC can be detected via this technique [17]. In this study, we followed the standard procedure using a probe provided by the manufacturer (Guang Zhou LBP Medicine Science & Technology Co., Ltd., Guang Zhou, China). The results were analyzed by a specialized staff member from the Key Laboratory of Stem Cell Transplantation in Ningbo. The personnel used the related probe manual to interpret the results. Based on previous reports, IHC was performed using standard techniques. The mean optical density was used to determine the difference in protein expression between BC patients and HC. Three random regions of each sample were selected, and the average option density (AOD) was calculated by measuring the integrated optical density (IOD) and the area of each region, which reflects the concentration of the target protein per unit area.

### Single-cell RNA Sequencing and primary analysis of sequencing data

Single-cell suspensions of 1 × 10^5^ cells/mL were prepared in PBS (HyClone). These single-cell suspensions were then loaded onto microfluidic devices, and scRNA-seq libraries were constructed according to the Singleron GEXSCOPE^®^ protocol using the GEXSCOPE^®^ Single-Cell RNA Library Kit (Singleron Biotechnologies) [18]. Individual libraries were diluted to 4 nM and pooled for sequencing on an Illumina HiSeq X platform with 150 bp paired-end reads. After scRNA-seq, fast-QC and fastp were used to remove low-quality raw reads and adaptor sequences. The reads were subsequently mapped onto the reference genome GRCh38 (Ensembl version 92 gene annotation) with STAR. Finally, gene counts and UMI counts obtained by FeatureCounts software were used to construct the expression matrix files.

### Statistical analysis

The median and interquartile range (IQR) of the marker levels were calculated based on the distribution of several markers (see the Additional file 1 The concentrations of the serum and urinary supernatant markers were analyzed in NPX units (log2-transformed normalized protein expression). All outlier samples were eliminated by protein interaction network and weighted gene coexpression network analysis (WGCNA). Differential protein analysis was performed for the serum and urine supernatants between the BC group and the HC group. Gene Ontology (GO) and Kyoto Encyclopedia of Genes and Genomes (KEGG) pathway enrichment analyses were performed for the differentially expressed proteins [19, 20]. The differences between BC and HC were analyzed by Student’s t test (2-tailed test). The expression of potential biomarkers was confirmed by IHC and single-cell sequencing. A logistic regression model was constructed to analyze the significantly differentially expressed protein markers, and LASSO was used to construct a model of serum concentration, urine supernatant, and the combination of serum and urine supernatant.

The receiver operating characteristic (ROC) curves of multiple LASSO models were analyzed for the BC group and the HC group. The ROC curves of the following variables were generated: (1) combined serum PEA biomarkers; (2) combined PEA biomarkers in the urine supernatant; and (3) combined PEA biomarkers in the serum and urine supernatants. The sensitivity, specificity, accuracy, PPV, and NPV of the LASSO model and the FISH assay for BC were determined. All the data were expressed as a single variable. Positive and negative results for the model were determined by cutoff values, whereas positive and negative values for FISH were determined by clinical reports. The chi-square test was used for statistically analyzing the model and FISH data. All tests were two-sided, and all differences were considered to be statistically significant at P < 0.05. All the statistical analyses were performed using R (version 3.6.1) and SPSS (version 26.0); all the graphs were constructed using the ggplot2 package.

## Results

### Baseline characteristics of the participating population

In total, samples from 99 patients with BC and 50 HC were collected from the Department of Urology, The First Affiliated Hospital of Ningbo University, China. The baseline characteristics of the participants are listed in Table 1, and additional detailed clinical features can be found in the Additional file materials. Serum and urine supernatant samples were evaluated using the Olink Oncology II Panel. All 92 biomarkers were detected in the serum and urine supernatants of more than 95% of the participants and were used for further analysis. To minimize the effect of outliers on the results, all outliers were excluded by WGCNA (Additional file 1: Figs. S1, S2). In the final data, 97 urine supernatant samples were obtained from the BC group (M/F = 79/18; mean age = 67.9 ± 11.8 years), and 50 samples were obtained from the HC group (M/F = 36/14; mean age = 50.8 ± 18.0 years). Additionally, 99 serum samples were included from the BC group (M/F = 80/19; mean age = 67.4 ± 12.2 years), and 47 samples were included from the HC group (M/F = 35/13; mean age = 50.5 ± 17.8 years).

**Table 1:** Summary of the characteristics of the BC patients and HC

Variables	–	No.	Total no.
BC group	–	–	–
Age(year)	Mean(± SD)	67.46(± 12.18)	–
Gender	Male	80	99
–	Female	19	–
Smoking history	Non-smoker	85	99
–	Smoker	14	–
Number of tumors	Single tumor	46	99
–	Multiple tumors	53	–
Histological grade	PUNLMP	5	99
–	Low grade	38	–
–	High grade	56	–
Tumor size	< 15 mm	26	99
–	30 > Size ≥ 15 mm	33	–
–	≥ 30 mm	40	–
TNM stage	NMIBC	79	99
–	MIBC	20	–
–	–	–	–
Healthy control	–	–	–
Age(year)	Mean(± SD)	50.78(± 19.94)	
Gender	Male	36	50
–	Female	14	–

*BC* bladder cancer, *PUNLMP* Papillary urothelial neoplasm of low malignant potential, *HC* healthy control

### Differential proteins in the urine supernatant and serum

**Fig. 2:**
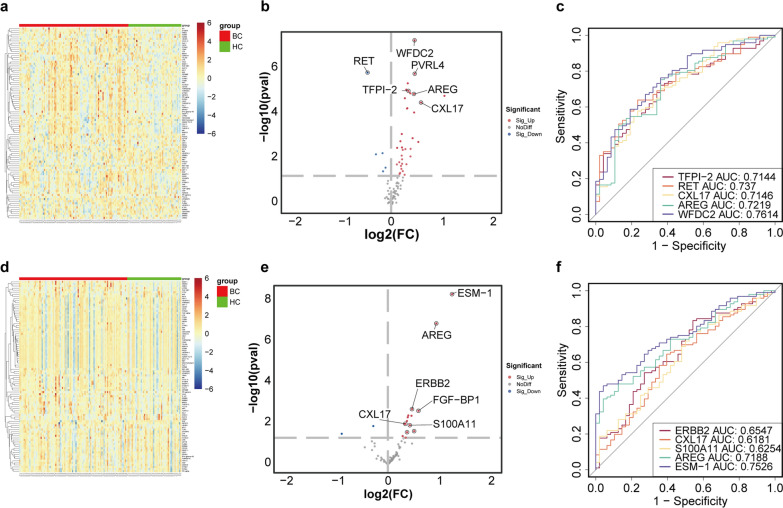
Volcano plots of the differential expression of all the proteins detected in the serum and urine supernatant samples and ROC curves of the differentially expressed proteins. **a** The overall serum data. **b** In the serum, significant differences in biomarkers occurred between the BC and HC. **c** ROC curves of individual biomarkers in serum. **d** The overall data of the urine supernatant. **e** In the urine supernatant, significant differences in biomarkers occurred between the BC and HC. **f** ROC curves of individual biomarkers in the urine supernatant; P < 0.05, two-tailed t test

A heatmap was generated to visualize the overall data, which represented the expression levels of 92 proteins in the serum and urine supernatant samples (Fig. 2a, d). Volcano plots were constructed to show P values and fold changes for biomarkers that were statistically significant in the differential expression analysis (Fig. 2b, e).

In the urine supernatant, there were 17 significantly differentially expressed biomarkers (p < 0.05) (including 15 upregulated and two downregulated biomarkers) between the BC and HC samples. We conducted the Kolmogorov‒Smirnov (KS) test and found that the NPX values of most proteins in the BC and HC samples were approximately normally distributed. We determined Spearman’s correlation coefficient to quantify the correlation between the biomarkers in the two groups. According to the area under the ROC curve (AUC), the five biomarkers in the urine supernatant were S100A11 (AUC = 0.625), ERBB2 (AUC = 0.655), CXL17 (AUC = 0.618), AREG (AUC = 0.719), and ESM-1 (AUC = 0.753) (Fig. 2f).

In the serum samples, 40 significantly differentially expressed biomarkers were identified (including 35 upregulated and five downregulated biomarkers) between the BC and HC groups (p < 0.05). According to the area under the curve (AUC), the five biomarkers in the serum were RET (AUC = 0.737), TFPI-2 (AUC = 0.714), CXL17 (AUC = 0.715), AREG (AUC = 0.722), and WFDC2 (AUC = 0.761) (Fig. 2c).

### GO and KEGG pathway enrichment analysis

To investigate the enrichment pathways and potential functions of the differentially expressed proteins associated with BC, we performed GO and KEGG analyses. The GO terms included molecular functions (MF), biological processes (BP), and cellular components (CC) [19].

**Fig. 3:**
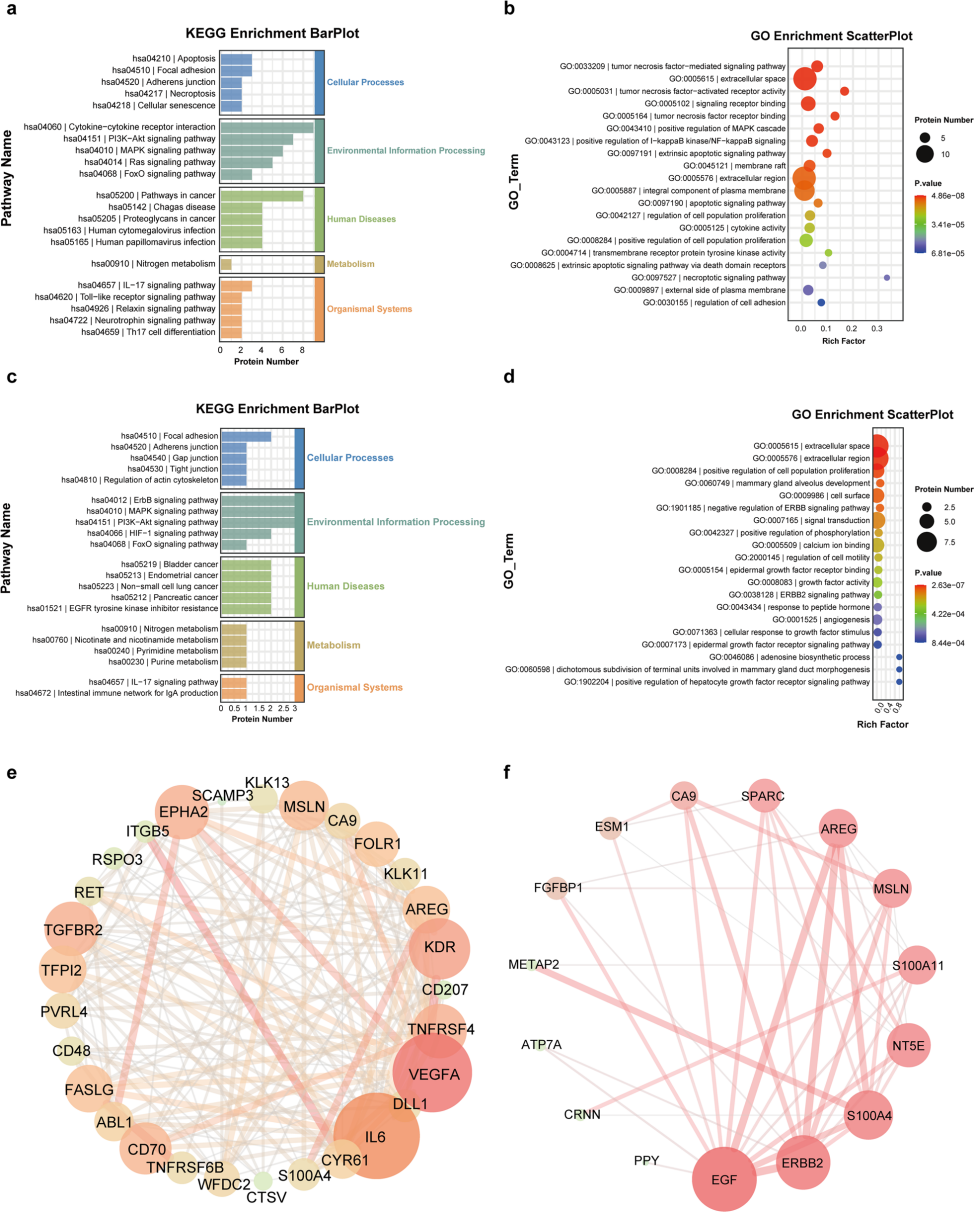
Proteins with significant differences were subjected to GO and KEGG enrichment analyses. **a** KEGG enrichment analysis of urinary supernatant biomarkers (bar plot). **b** GO enrichment analysis of urinary supernatant biomarkers. **c** KEGG enrichment analysis of the serum biomarkers (bar plot). **d** GO enrichment analysis of the serum biomarkers. PPI network analysis of **e** serum and **f** urine supernatants

The results of the GO analysis of the serum samples showed that the proteins were enriched mainly in BP, such as tumor necrosis factor-mediated signaling pathways, positive regulation of the MAPK cascade, positive regulation of I-kappaB kinase/NF-kappaB signaling, and regulation of cell proliferation. Evaluation of cellular localization revealed that most of the proteins were localized in the extracellular region and on the cell membrane, and some of them were present at both sites. The results of the KEGG analysis showed that the enriched pathways included mainly the PI3K-Akt signaling pathway, MAPK signaling pathway, and Ras signaling pathway (Fig. 3c, d).

The results of the GO analysis of the urine supernatant showed that the genes enriched in BP were positive regulators of cell proliferation and negative regulators of the ERBB signaling pathway. Assessment of cellular localization revealed that the proteins were localized in extracellular regions and the cell membrane. The results of the KEGG analysis showed that the proteins in the urine supernatant were enriched mainly in the ErbB signaling pathway, MAPK signaling pathway, HIF-1 signaling pathway, and PI3K-Akt signaling pathway (Fig. 3a, b).

### Protein‒protein interaction network analysis

We performed a protein‒protein interaction (PPI) network analysis to elucidate the potential interactions between protein biomarkers obtained from the analysis of serum and urine supernatant samples and to identify key proteins among them. We did not find any of the top five serum biomarkers based on the AUC in the PPI network (Fig. 3e). In contrast, IL6 and VEGFA had the highest degree scores, suggesting that they might play an important role in BC. Additionally, IL6 and VEGFA were closely associated with AREG, TFPI-2, and RET. Among the differentially expressed proteins in the urine supernatant (Fig. 3f), EGF and ERBB2 had the highest degree scores, suggesting that they might also play a role in BC. These two proteins were associated only with AREG.

### Correlations of differentially expressed proteins with clinical features

**Fig. 4:**
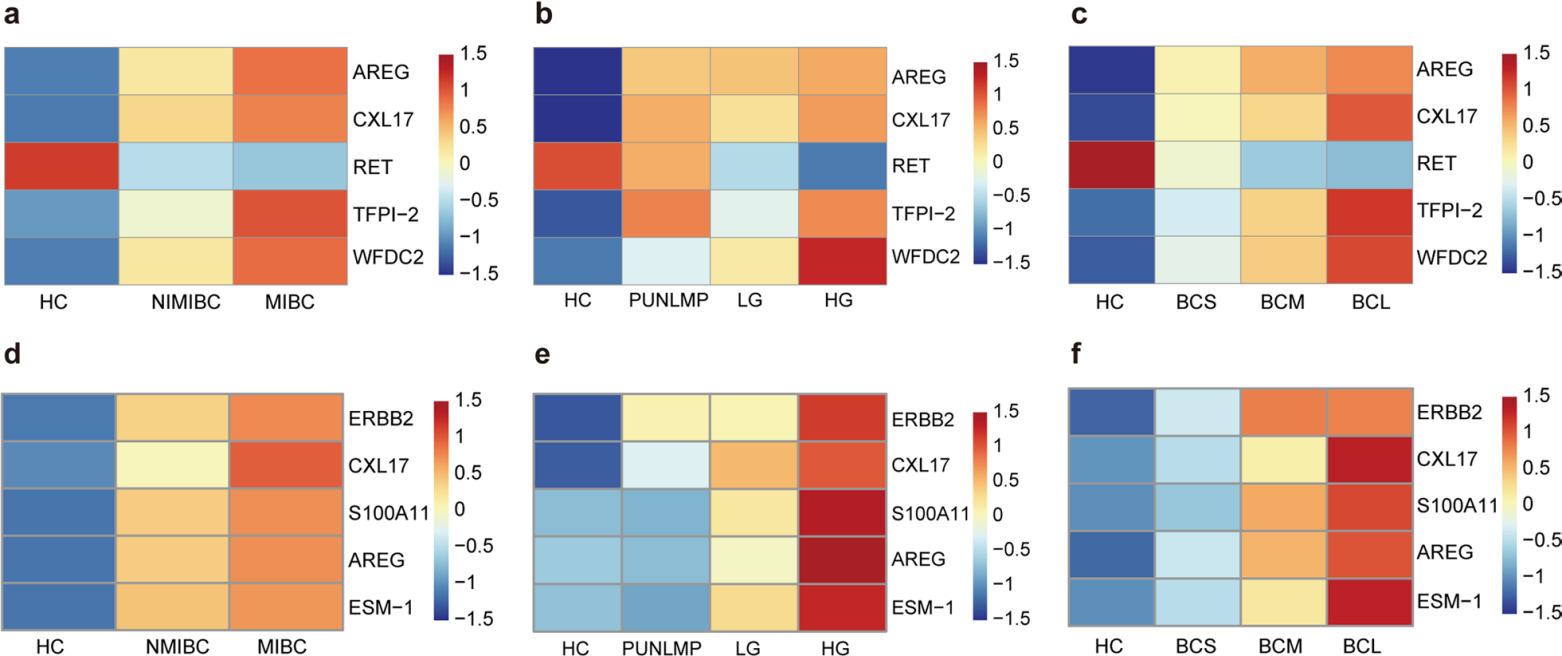
Changes in the serum protein markers according to **a** stage, **b** grade, and **c** tumor size. Changes in protein markers in the urine supernatant with **d** stage, **e** grade, and **f** tumor size

We determined the correlation between the previously obtained differentially expressed proteins and the clinical characteristics of BC patients. The results showed that the expression of AREG, CXL17, TFPI-2, and WFDC2 in the serum was correlated with the stage of BC. The expression of these proteins was significantly greater in advanced BC stages than in early BC stages (Fig. 4a). The expression of WFDC2 was greater in high-grade BC patients than in low-grade patients (Fig. 4b). The expression of TFPI-2 and WFDC2 was greater in large tumors than in small tumors. The expression of RET was the highest in normal controls, and RET was downregulated in terms of stage, grade and tumor size in BC patients (Fig. 4c).

In the urine supernatant, the expression of ERBB2, CXL17, S100A11, AREG, and ESM-1 increased with increasing stage (Fig. 4d). This pattern was also observed for BC grade, where the expression of ESM-1, S100A11, and AREG was significantly greater in high-grade BC patients than in low-grade patients (Fig. 4e). A higher expression of these five proteins was also associated with a larger tumor size (Fig. 4f).

### Expression validation and IHC of key biomarkers

We identified six potential protein markers for BC based on the maximum fold change and p values between the BC group and the HC group. These proteins included WFDC2, PVRL4, and RET in serum and ESM-1, AREG, and FGFBP1 in the urine supernatant.

**Fig. 5:**
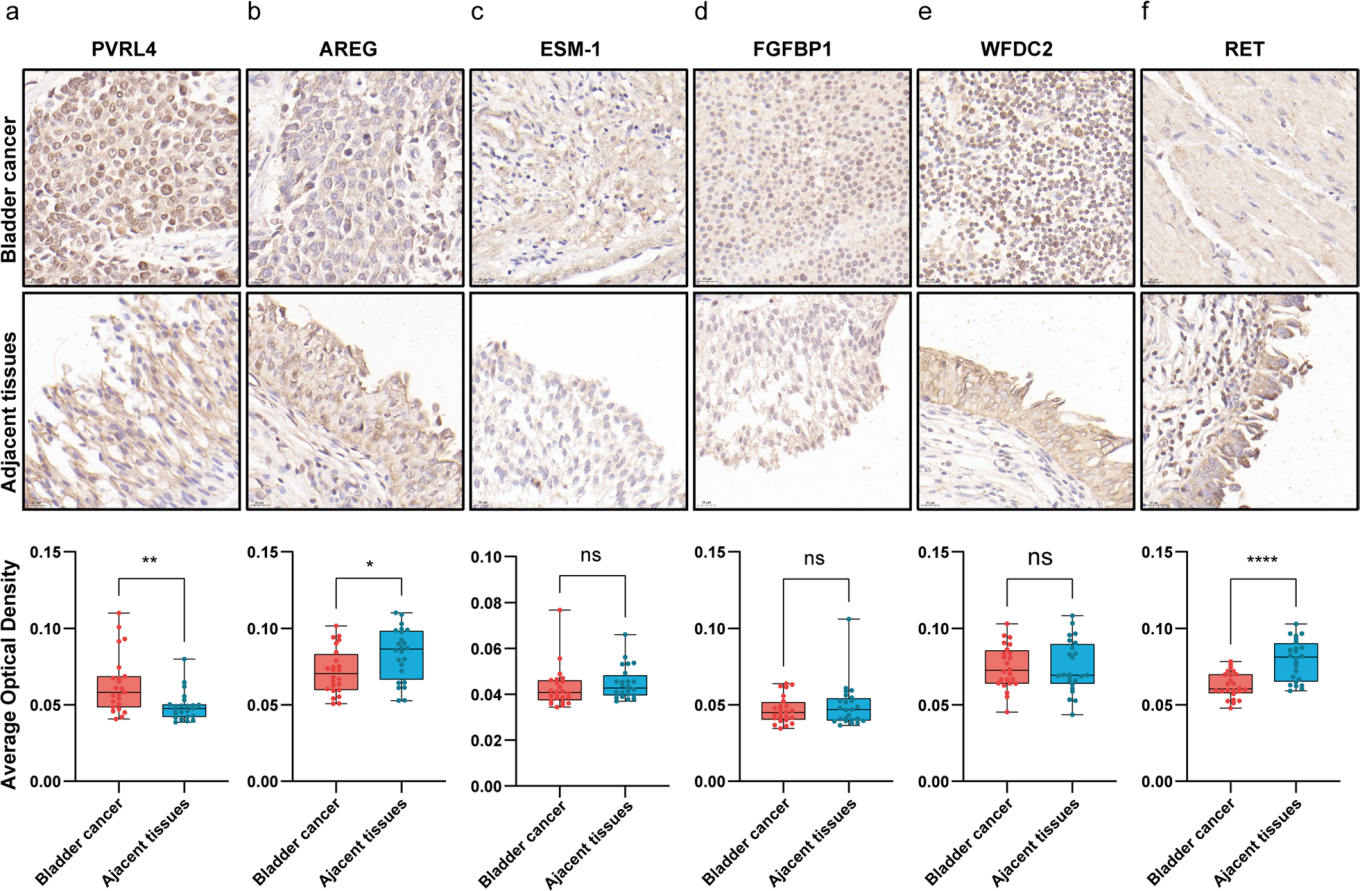
The results of immunohistochemical analysis of potential biomarkers of bladder cancer. **a**–**f** Immunohistochemical analysis of potential biomarkers for bladder cancer and adjacent tissues; ns: p ≥ 0.05, * p < 0.05, ** p < 0.01, and **** p < 0.0001 (two-tailed t test)

We performed IHC on cancerous and adjacent tissues collected from 24 BC patients, each with paired samples. The results of IHC showed that these six proteins were expressed in BC (Fig. 5). PVRL4 is a membrane protein that is more highly expressed in cancerous tissues than in adjacent tissues (P < 0.01). AREG was localized in the endoplasmic reticulum, extracellular region, and nucleus and was expressed at slightly greater levels in adjacent tissues than in cancer tissues (P < 0.05). RET is also a membrane protein that is expressed at significantly lower levels in cancer tissues than in normal tissues (P < 0.0001). ESM-1, WFDC2, and FGFBP1 were distributed within cells and within the cell membrane, but the difference in their expression between adjacent and cancerous tissues was not significant (Fig. 5a–f).

**Fig. 6:**
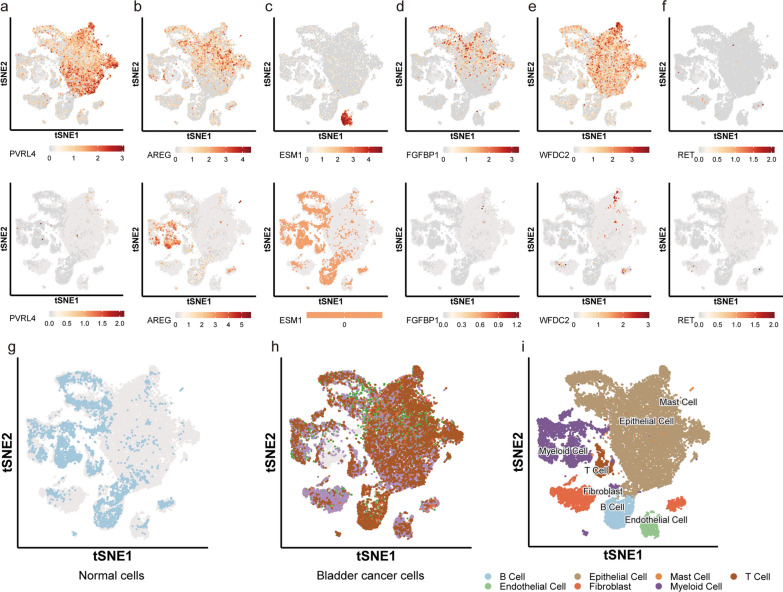
Results of single-cell histology of potential biomarkers for bladder cancer. **a**–**f** The expression of potential biomarkers in the urinary supernatant of bladder cancer patients and adjacent tissues. **g**–**i** Sample classification of bladder cancer tissue and adjacent tissue

Next, we examined the expression of the above proteins in the BC and HC groups at the transcriptome level using single-cell transcriptome sequencing (Fig. 6). The sequencing results revealed that the BC were B cells, epithelial cells, endothelial cells, mast cells, T cells, fibroblasts, and myeloid cells. Myeloid cells, B cells, T cells, a few epithelial cells, and a few fibroblasts were identified in the HC samples (Fig. 6i). The results of the single-cell sequencing analysis were similar to those of PEA and IHC and confirmed that the above six genes were expressed at the mRNA level in the BC samples. PVRL4 was predominantly expressed in BC epithelial cells, AREG was expressed in BC epithelial cells and normal control myeloid cells, and FGFBP1 and WFDC2 were expressed in bladder cancer epithelial cells but not in normal tissue. ESM-1 was specifically expressed in BC endothelial cells, whereas RET was not expressed in BC or normal tissue (Fig. 6a–f).

### Multivariate diagnostic performance of PEA for detecting proteins

We used LASSO regression to generate combinations of biomarkers that could distinguish between the BC group and the HC group. This approach reduced the number of features by decreasing the coefficients of some relevant variables to zero. We selected the optimal model based on the minimum binomial deviance between the multinomial logistics regression model and group categories. We also selected the simplest model that had an accuracy similar to that of the optimal model by choosing the shrinkage parameter (λ_min_) at 1 standard error (λ_min_ + 1SE) from the optimal model to avoid overfitting. We performed 50 replicates of a fivefold cross-validation analysis and used AUC scores and accuracy as the metrics for model selection. Then, we evaluated the performance of the model for different stages and grades of cancer in the BC group.

**Fig. 7:**
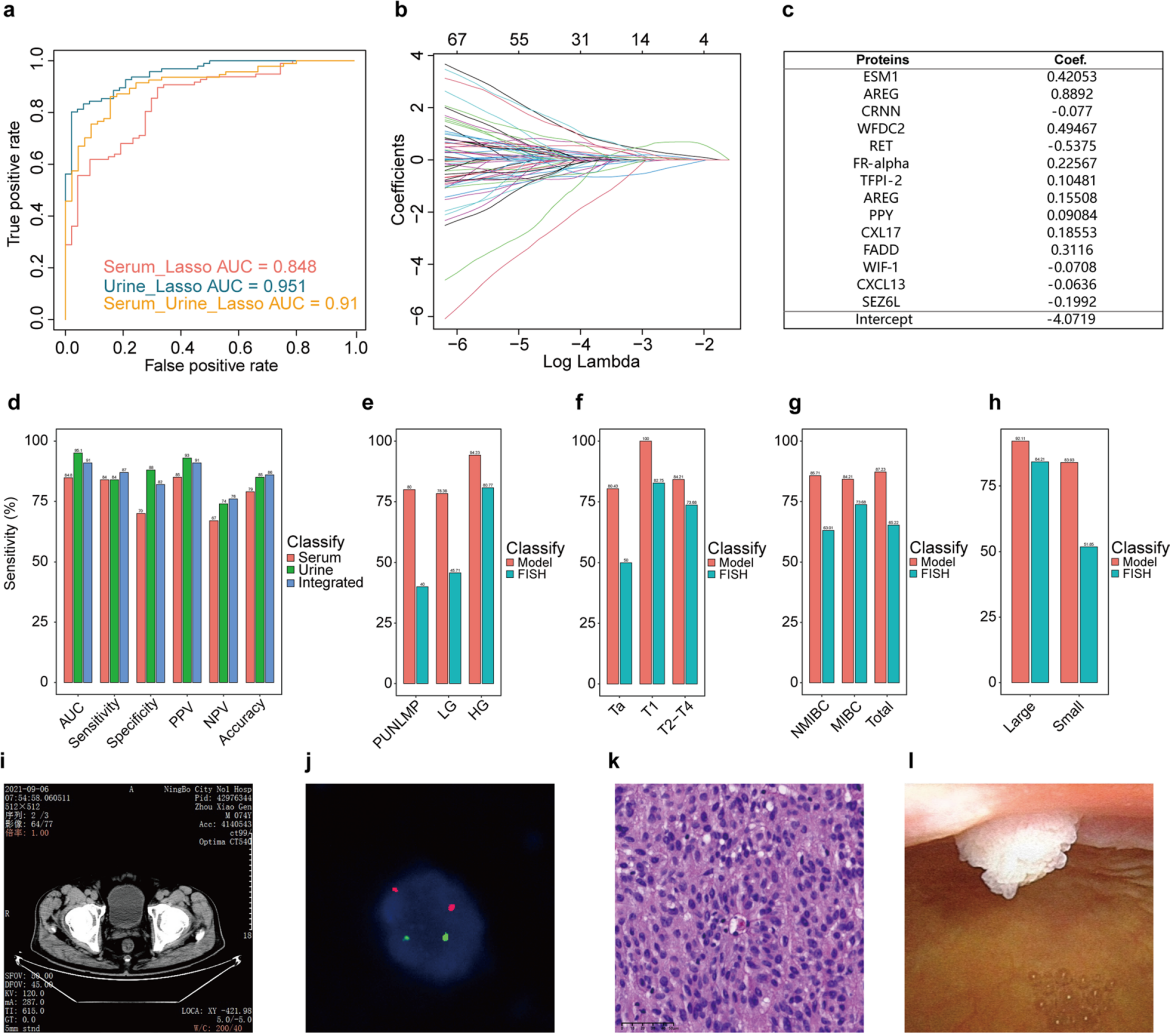
Comparison between the results of the prediction model and FISH. **a** The area under the curve of the serum and urine supernatant integration model. **b** The number of features that the model features changes with the shrinkage parameter. **c** The characteristics of the proteins and coefficient sizes in the model. **d** Comparison of the AUC, sensitivity, specificity, PPV, NPV, and accuracy among serum, urine supernatant, and integrated models. **e** Comparison between the sensitivity of the integration model and that of FISH for PUNLMP, LG, and HG tumors. **f** Comparison between the sensitivity of the integration model and that of FISH at the Ta, T1, and T2-T4 stages. **g** Comparison between the sensitivity of the integrated model and FISH in NMIBC, MIBC, and all other patients. **h** Model comparison of FISH for tumor size; large (≥ 30 mm) and small (< 30 mm). **i**–**l** Enhanced CT, FISH, tissue sectioning, and cystoscopy findings in typical patients with small tumors (< 30 mm); p < 0.05; chi-square test

We first performed LASSO regression analysis using the data on serum samples from the BC group and the HC group. The model included nine proteins (WFDC2, RET, FR-alpha, TFPI-2, AREG, PPY, CXL17, FADD, and WIF-1) and had an AUC of 0.848 (95% CI: 0.7837–0.9132), an accuracy of 0.79, a sensitivity of 0.84, and a specificity of 0.70 (Fig. 7d).

We also performed LASSO regression analysis using the data from urine supernatant samples from both groups and identified 21 proteins out of 92 protein markers (ESM-1, AREG, ERBB2, FGF-BP1, CAIX, CXL17, EGF, CRNN, WISP-1, CTSV, KLK13, TLR3, CD27, ABL1, ANXA1, hK8, IFN-gamma-R1, ADAM 8, VIM, GPC1, and TNFRSF4). This biomarker combination had an AUC of 0.951 (95% CI: 0.919–0.9821), an accuracy of 0.85, a sensitivity of 0.84, and a specificity of 0.88.

To improve the efficacy of the diagnosis, we combined urine supernatant and serum samples from the same participants in the two groups, and independent samples were excluded during the data screening process. We constructed an integrated model of urine supernatant and serum consisting of 14 protein markers, ESM-1, AREG, and CRNN, from urine samples and WFDC2, RET, FR-alpha, TFPI-2, AREG, PPY, CXL17, FADD, WIF-1, CXCL13, and SEZ6L from serum samples. The AREG protein, which is upregulated in various cancers, was present in the urine supernatant and serum of the integrated model. The diagnostic performance of the biomarker combination is shown in Fig. 7a–c. The combination had an AUC of 0.91 (95% CI: 0.8612–0.9582), an accuracy of 0.86, a sensitivity of 0.87, and a specificity of 0.82 for distinguishing between the two groups.

All the protein markers in the combined model were also present in both the urine supernatant model and the serum model. The combined model had fewer features than the urine supernatant model alone (14 vs. 21), which decreased the risk of overfitting. The combined model also had higher accuracy than the serum model alone (0.86 vs. 0.79), but its accuracy was not significantly different from that of the urine supernatant model (0.86 vs. 0.85). The sensitivities of the three models were similar (0.84, 0.84, and 0.87). The specificity of the combined model was slightly lower than that of the urine supernatant model (0.82 vs. 0.88) (Fig. 7d). We selected a model with high accuracy, a low number of features, and high sensitivity. Therefore, we used the combined LASSO model consisting of three urine supernatant biomarkers and 11 serum biomarkers for all subsequent analyses.

### Performance of multivariate diagnostic models compared to that of FISH

FISH is a conventional method for detecting BC. To compare the performance of the combined model and FISH, further analyses were performed based on the different stages and grades of BC. In terms of performance in differentiating between BC and HC, the overall sensitivity of the integrated model (87.23%) was significantly greater than that of FISH (65.22%) (p < 0.05).

Evaluation of the model in BC patients stratified by grade showed that the model outperformed FISH in patients with high-grade tumors (94.23% vs. 80.77%), but the difference in performance between the methods was not significant. In patients with low-grade tumors, the sensitivity of the model was almost twice that of FISH (78.38% vs. 45.71%) (p < 0.05). In this study, five patients had PUNLMP; four of these patients (80%) were detected by the model, whereas FISH could only detect two of them (40%) (Fig. 7e). The sensitivity of the model (85.71%) for detecting NMIBC patients was significantly greater than that of FISH (65.22%) (p < 0.05). The sensitivity of the model for detecting MIBC patients was also significantly greater than that of FISH (84.21% vs. 73.68%) (p < 0.05).

When the data were grouped by the TNM stage of BC patients, we found that the model had a significantly greater detection rate than FISH in patients with stage Ta disease (80.43% vs. 50%) (p < 0.05). Our model showed a sensitivity of 100% in detecting patients with stage T1 disease, which was significantly greater than the sensitivity of FISH (82.75%) (Fig. 7f).

We also evaluated the performance of the model in detecting tumors smaller than 30 mm. The model had a sensitivity of approximately 92.11% for detecting larger tumors (≥ 30 mm) and approximately 83.93% for detecting smaller tumors (< 30 mm). In contrast, FISH had a decreased ability to detect larger and smaller tumors. In the subgroup of tumors < 30 mm, the sensitivity of FISH was only 51.9%, which was significantly different from the sensitivity of the model (Fig. 7 h) (p < 0.05). The results showed that the model performed better than FISH did. This patient had a small tumor in the parietal wall of the bladder, but the enhanced CT images did not reveal any tumor. The results obtained using FISH were negative, whereas those obtained using our model were positive (Fig. 7i). This case highlights the utility of this approach. The lesion in the parietal wall was very small and was subsequently diagnosed as a low-grade Ta carcinoma. These findings showed the advantages of the model, especially for detecting low-grade small lesions and early BC.

Overall, the model had higher sensitivity for detecting larger tumors. The sensitivity was lower for smaller tumors, lower grade tumors, and NMIBC stage tumors, but it was still higher than the sensitivity of FISH. The smallest tumor that the model detected had a diameter of approximately 2 mm, which showed that it was better than FISH.

## Discussion

BC is the most common malignancy in the urinary tract. It generally has a poor prognosis and substantially decreases quality of life [1]. Early diagnosis can greatly improve patient survival and quality of life while reducing patient burden. Liquid biopsy is a feasible method for the early diagnosis of BC. It is noninvasive, highly reproducible, and can be used for the early diagnosis and treatment of BC [21]. As the molecular changes that occur during tumor development are extremely complex, a combination of multiple diagnostic features can perform considerably better than a single diagnostic marker. In this study, we identified potential inflammation-related protein biomarkers of BC using Olink PEA proteomics.

We applied a new liquid biopsy technique known as PEA, which combines the advantages of antibody-based and DNA-based assays. PEA is highly sensitive and specific, and it can detect low protein levels in small sample volumes. We used PEA to detect 92 inflammation-related proteins in BC patients and HC participants. We identified five proteins with the highest AUC in the differential analysis of serum and urine supernatants and evaluated their relationship with clinical features. All the proteins except RET were positively correlated with BC stage, grade, and size. AREG, ESM-1, FGFBP1, WFDC2, PVRL4, and RET are potential protein markers for BC.

We found that the expression of RET was significantly lower in BC patients. RET is a tyrosine kinase receptor that is primarily expressed in tissues of the nervous system, adrenal gland, and thyroid gland. It acts with the endogenous ligand GDNF (glial cell-derived neurotrophic factor) [22]. RET can also act as an oncogene and participate in the development and progression of several human cancers [23]. In this study, RET expression was lower in the serum and urine supernatants of patients with BC than in those of healthy individuals, as determined by single-cell sequencing and IHC. Two major isoforms of RET, RET9, and RET51, are expressed in papillary thyroid carcinoma (PTC), but information on the level of RET expression is lacking [23]. Additionally, no published information is available on the expression of RET in BC. In this study, we used PEA technology to detect aberrant RET protein expression in BC.

The protein AREG is a ligand for the epidermal growth factor receptor (EGFR), which is a widely expressed transmembrane tyrosine kinase [24]. One study considered AREG a prognostic marker for BC patients and revealed a strong correlation between the survival of BC patients and the expression of AREG mRNA [25]. Increased expression of AREG can potentially enhance cell growth and angiogenesis, thereby promoting tumor progression and reducing overall survival in patients with BC [25]. The PEA results showed that the expression of AREG increased with the grade and stage of BC, and its presence in BC was determined by single-cell sequencing and IHC. Although the IHC results did not show satisfactory statistical results, we speculated that this might be an error due to the small sample size. Overall, our findings suggested that AREG is a promising protein that might be further investigated for its specificity toward BC.

Poliovirus receptor-like (PVRL4) is a type I membrane protein that is expressed at significantly greater levels in BC tissue than in normal tissue. Moreover, PVR-4 can promote cancer cell proliferation and metastasis. It can also interact with the tyrosine kinase receptor ERBB2 to activate it. This, in turn, stimulates the PI3K-AKT pathway to promote tumor proliferation and metastasis [26]. PVRL4 is a tumor-associated antigen found on the surface of most urothelial carcinoma cells. Enfortumab vedotin, an antibody‒drug conjugate (ADC) targeting PVRL4, was developed and approved by the FDA for treating locally advanced and metastatic urothelial cancer [26]. Our finding that PVRL4 expression is elevated in BC patients was consistent with the results of other studies, suggesting that PVRL4 can be used for diagnosing BC.

The protein WFDC2, also known as HE4 (human epithelial cell protein 4), is a protease inhibitor involved in the innate immune defense of the respiratory tract and nasal cavity [27]. High levels of WFDC2 have also been reported in the early stage of BC [27]. A study showed that serum levels of WFDC2 are high in patients with BC in the urinary tract but not in patients with different TNM stages [28]. We found that the serum WFDC2 concentration was high in BC patients and increased with stage, grade, and size. The results of single-cell transcriptome sequencing also showed greater expression of WFDC2 in the BC group than in the healthy control group at the transcriptome level; the expression of WFDC2 in BC was detected by IHC. FGFBP1 is a secreted protein that specifically binds to and promotes the release of extracellular matrix-anchored fibroblast growth factors (FGFs) [29]. It can induce the tumorigenic potential of epithelial cells and is highly expressed in oral cancer cell lines and tissues [30]. FGFBP1 might be a promising biomarker for predicting the prognosis of BC patients treated with intravesical BCG [31]. We also found high levels of FGFBP1 in the serum and urine supernatants of BC patients, and single-cell sequencing showed similar results. The evidence for biomarkers as predictors of BC is not strong. ESM-1 (endothelial cell-specific molecule) has angiogenic and inflammatory properties and might affect vascular permeability [32]. It is highly expressed in the blood vessels of invasive BC tissues [33]. Higher levels of ESM-1 in serum and urine supernatants were reported in BC patients than in HC[34], which matched our findings. This increase is associated with an increase in the viability, migration, and invasion of BC cells and the inhibition of their apoptosis [33].

We developed a comprehensive diagnostic model of 14 biomarkers in serum and urine supernatants using the LASSO method; we recorded an AUC of 0.91, an accuracy of 0.86, a sensitivity of 0.87, and a specificity of 0.82. Compared to FISH, our model was better at detecting BC cases of different grades, stages, and sizes. Our model outperformed FISH in identifying low-risk BC (including low-grade and low-stage BC) and could accurately identify tumors smaller than 30 mm. The sensitivity of our model was approximately 30% greater than that of FISH (83.93% vs. 51.8%). A study established a protein signature for seven biomarkers (ANG, APOE, IL8, MMP9, MMP10, PAI-1, and VEGFA) that could differentiate between BC patients and non-BC patients. The method used in that study had an AUC of 0.88 and a sensitivity of 74%.[35] Another study used A1TA to distinguish between BC patients and HC. The study recorded an AUC of 0.82 and a sensitivity of 74% [36]. Our model had a higher detection sensitivity than FISH and previous protein marker assays, especially for early, low-grade, and small tumors, indicating that it might be useful for the early diagnosis of BC.

Our study has several limitations. First, the sample size was relatively small, and a larger sample size is needed for training and validation of the model. Second, the diagnostic model was constructed for point analysis without long-term follow-up data. Therefore, relating false positives to events that occurred during follow-up was not possible. We aimed to increase the sample size to include additional patients with benign bladder disease and conduct follow-up studies to obtain more reliable results.

## Conclusion

To summarize, we used Olink PEA technology to measure 92 cancer-related proteins in the serum and urine of BC patients and healthy control participants. We identified 40 differentially expressed proteins in the serum and 17 differentially expressed proteins in the urine between the groups. The proteins in the serum and urine supernatants were mainly enriched in BPs; most of the proteins were located in extracellular regions and on the cell membrane. The MAPK pathway, Ras pathway, VEGF pathway, and PI3K/Akt pathway were the pathways associated with the enrichment of the differentially expressed proteins. Six potential BC biomarkers were identified by the PEA technique, of which RET was the first to be reported. These proteins need to be further investigated in BC patients because they might help elucidate the mechanism underlying the development of BC. Our first diagnostic model for BC, constructed using data on proteins from serum and urine supernatants, showed better performance than traditional diagnostic methods, particularly for diagnosing early-stage, low-risk, and small tumors. Thus, this model might be useful for clinical application in the future.

### Supplementary Information

**Additional file 1: Figure S1.** Sample clustering of abnormal values in serum by WGCNA detection, WGCNA, Weighted correlation network analysis. **Figure S2.** Sample clustering of abnormal values in urine by WGCNA detection, WGCNA, Weighted correlation network analysis. **Figure S3.** Quality control of PEA assay proteins. **a** IQR for detection of proteins in serum. **b** IQR for detection of proteins in urine, *QC* quality control, *IQR* interquartile range. **Table S1.** Information of 92 proteins detected using PEA technology. **Table S2.** Clinical Characteristics of Serum Samples. Clinical Characteristics of Urine Supernatant Samples.

## Data Availability

All the data in this study are shown in the manuscript, and Additional file materials, and unprocessed data are available from the corresponding author upon reasonable request.

## Abbreviations

BC: Bladder cancer
NMIBC: Non-muscle-invasive bladder cancer
MIBC: Muscle-invasive bladder cancer
PUNLMP: Papillary urothelial neoplasm of low malignant potential
AREG: Amphiregulin
RET: Rearranged during transfection
WFDC2: WAP four-disulfide core domain protein 2
FGFBP1: Fibroblast growth factor binding protein 1
ESM-1: Endothelial cell-specific molecule 1
PVRL4: Poliovirus receptor-related 4
LASSO: Least absolute shrinkage and selection operator
PEA: Proximity extension analysis
AOD: Average optical density
IOD: Integral optical density
IQR: Interquartile range
ROC: Receiver operating characteristic
