# Supplementary material for: Identification of novel protein biomarkers from the blood and urine for the early diagnosis of bladder cancer via proximity extension analysis
Source: J Transl Med. 2024 Mar 26;22:314. doi: 10.1186/s12967-024-04951-z (PMC10967215; doi:10.1186/s12967-024-04951-z)
Supplement: Supplementary file 1 — Additional file 1: Figure S1. Sample clustering of abnormal values in serum by WGCNA detection, WGCNA, Weighted correlation network analysis. Figure S2. Sample clustering of abnormal values in urine by WGCNA detection, WGCNA, Weighted correlation network analysis. Figure S3. Quality control of PEA assay proteins. a IQR for detection of proteins in serum. b IQR for detection of proteins in urine, QC quality control, IQR interquartile range. Table S1. Information of 92 proteins detected using PEA technology. Table S2. Clinical Characteristics of Serum Samples. Clinical Characteristics of Urine Supernatant Samples. [file 12967_2024_4951_MOESM1_ESM.docx]

Additional file materials

Identification of novel protein biomarkers from the blood and urine for the early diagnosis of bladder cancer via proximity extension analysis

**Author list**

Tong Kong^1^, Yang Qu^1^, Taowa Zhao^1^, Zitong Niu^1^, Xiuyi Lv^2^, Yiting Wang^1^, Qiaojiao Ding^1^, Pengyao Wei^1^, Jun Fu^3^, Liang Wang^4^, Jing Gao^3^, Cheng Zhou^2^, Suying Wang^5^, Junhui Jiang^2^, Jianping Zheng^1*^, Kaizhe Wang^1*^, Kerong Wu^2*^

**Affiliations**

^1^Ningbo Cixi Institute of BioMedical Engineering, Ningbo Institute of Materials Technology & Engineering, Chinese Academy of Sciences (CAS), Ningbo, 315300, PR China

^2^Department of Urology, Key Laboratory of translational Research for Urology of Ningbo City, Key Laboratory of Precision Medicine for Atherosclerotic Diseases of Zhejiang Province, The First Affiliated Hospital of Ningbo University (Ningbo First Hospital), Zhejiang, China

^3^LC-Bio Technology Co., Ltd, Hangzhou, China

^4^Olink Proteomics, Shanghai, China

^5^Ningbo Clinical Pathology Diagnostic Centre

**Address correspondence to:**

Kerong wu, MD, PhD

Department of Urology, Key Laboratory of translational Research for Urology of Ningbo City, Key Laboratory of Precision Medicine for Atherosclerotic Diseases of Zhejiang Province, The First Affiliated Hospital of Ningbo University (Ningbo First Hospital), Zhejiang, China

Email: [fyywukerong@nbu.edu.cn](mailto:fyywukerong@nbu.edu.cn)

Phone: (+86)–17757461301

**List of Additional file Tables**

Additional file Figure 1: Sample clustering of abnormal values in serum by WGCNA detection, WGCNA, Weighted correlation network analysis.

Additional file Figure 2: Sample clustering of abnormal values in urine by WGCNA detection, WGCNA, Weighted correlation network analysis.

Additional file Figure 3: Quality control of PEA assay proteins. (a) IQR for detection of proteins in serum. (b) IQR for detection of proteins in urine, QC, quality control; IQR, interquartile range.

Additional file Table 1. Information of 92 proteins detected using PEA technology

Additional file Table 2. Clinical Characteristics of Serum Samples

Additional file Table 3. Clinical Characteristics of Urine Supernatant Samples

## Additional file Figures


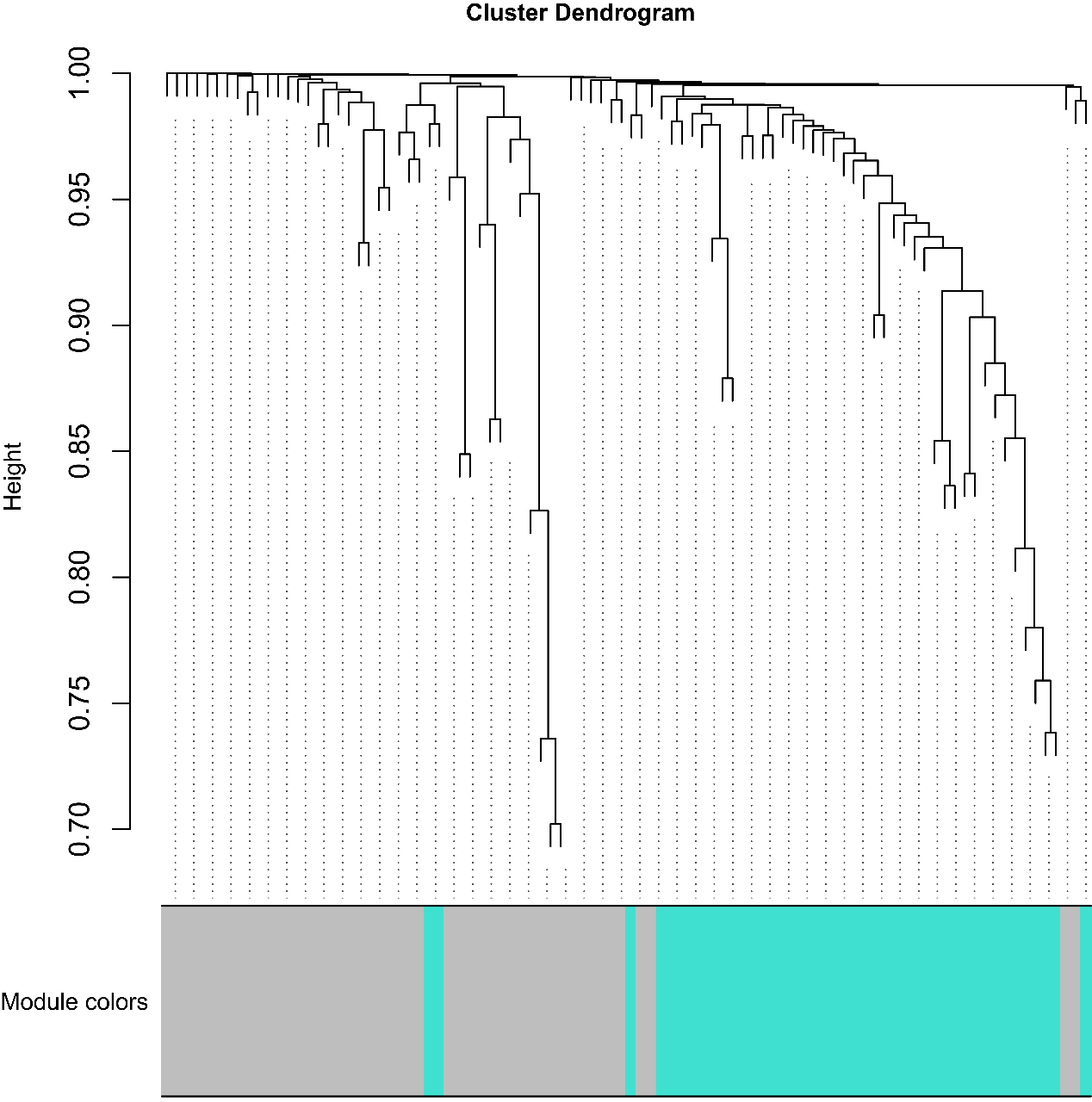


Additional file **Figure 1.** Sample clustering of abnormal values in serum by WGCNA detection, WGCNA, Weighted correlation network analysis.


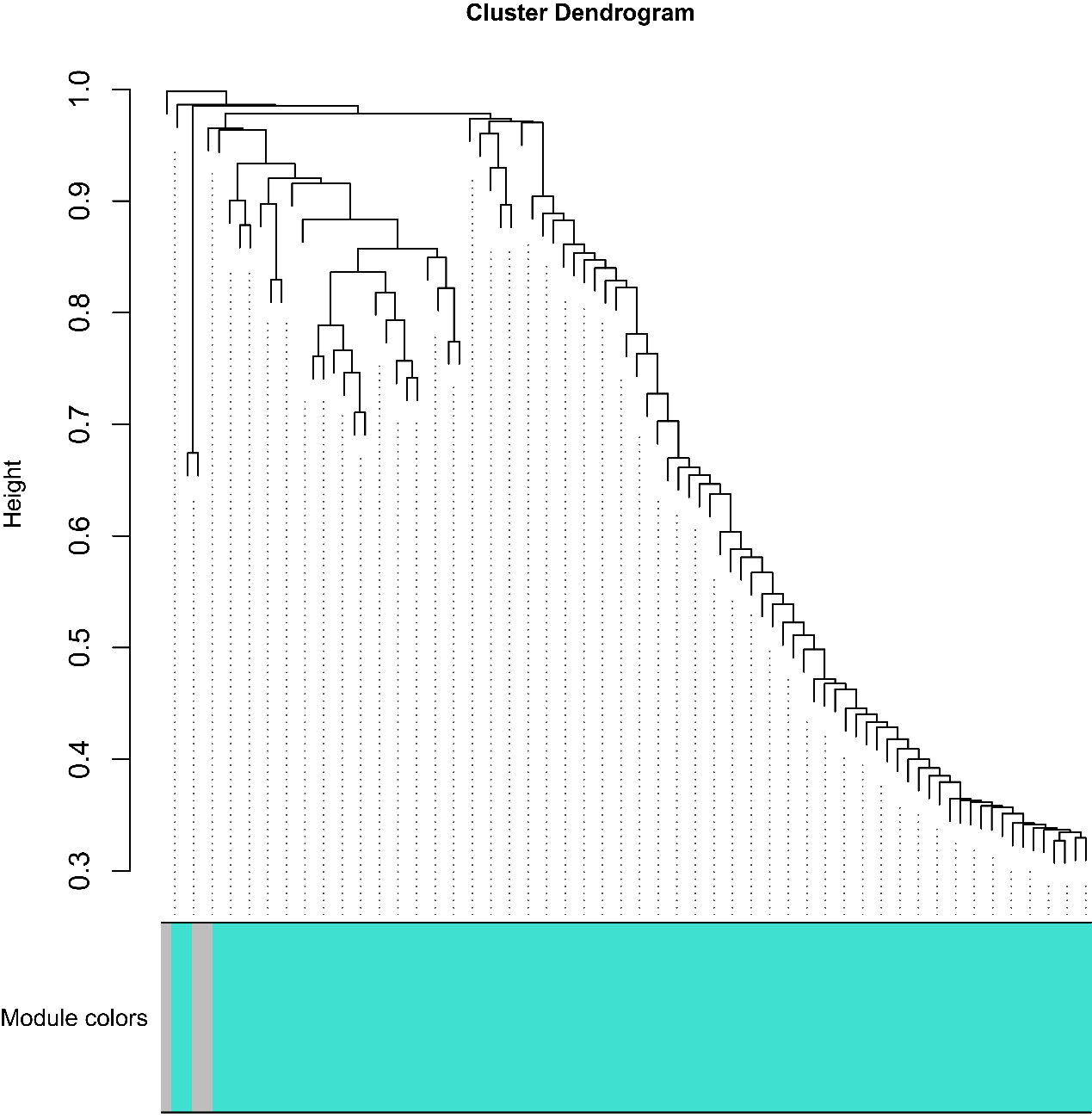


Additional file **Figure 2.** Sample clustering of abnormal values in urine by WGCNA detection, WGCNA, Weighted correlation network analysis.


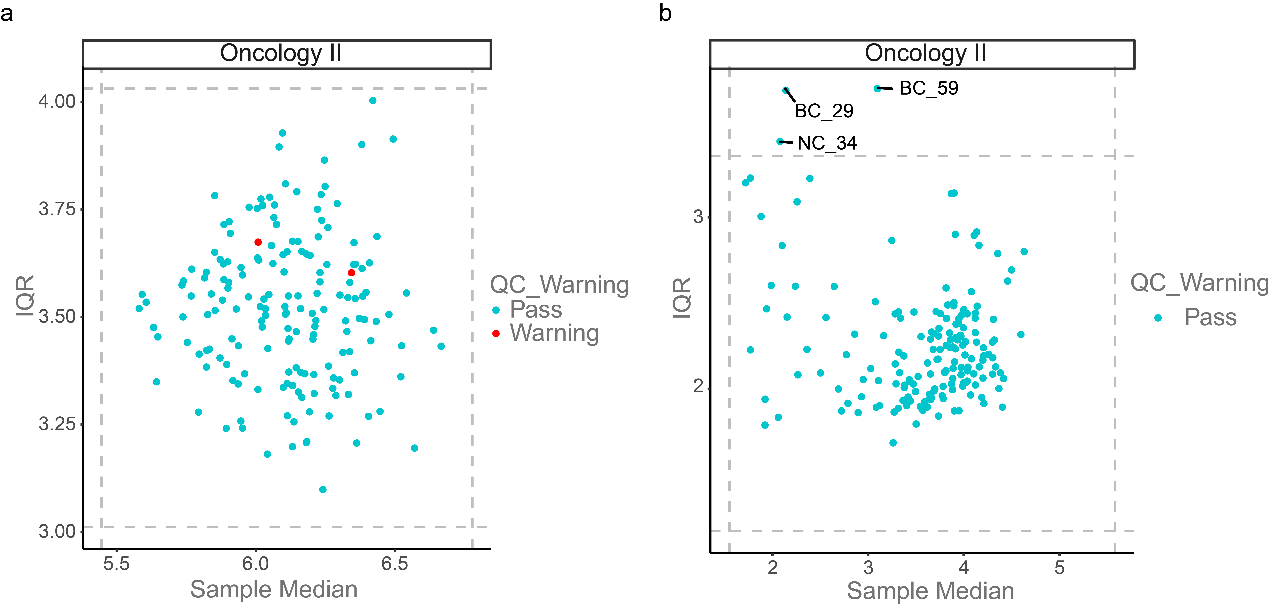


Additional file **Figure 3.** Quality control of PEA assay proteins. (a) IQR for detection of proteins in serum. (b) IQR for detection of proteins in urine, QC, quality control; IQR, interquartile range.

## Additional file table

Additional file **Table 1.** Information of 92 proteins detected using PEA technology

| **Protein name** | **UniProt ID** |
| --- | --- |
| 5'-nucleotidase (5'-NT) | P21589 |
| A disintegrin and metalloproteinase with thrombospondin motifs 15 (ADAM-TS 15) | Q8TE58 |
| Alpha-taxilin (TXLNA) | P40222 |
| Amphiregulin (AREG) | P15514 |
| Annexin A1 (ANXA1) | P04083 |
| Carbonic anhydrase IX (CAIX) | Q16790 |
| Carboxypeptidase E (CPE) | P16870 |
| Carcinoembryonic antigen (CEA) | P06731 |
| Carcinoembryonic antigen-related cell adhesion molecule 1  (CEACAM1) | P13688 |
| Cathepsin L2 (CTSV) | O60911 |
| CD27 antigen (CD27) | P26842 |
| CD160 antigen (CD160) | O95971 |
| CD48 antigen (CD48) | P09326 |
| CD70 antigen (CD70) | P32970 |
| Cornulin (CRNN) | Q9UBG3 |
| C-type lectin domain family 4 member K (CD207) | Q9UJ71 |
| C-X-C motif chemokine 13 (CXCL13) | O43927 |
| Cyclin-dependent kinase inhibitor 1 (CDKN1A) | P38936 |
| Delta-like protein 1 (DLL1) | O00548 |
| Disintegrin and metalloproteinase domain-containing protein 8 (ADAM8) | P78325 |
| Endothelial cell-specific molecule 1 (ESM-1) | Q9NQ30 |
| Ephrin type-A receptor 2 (EPHA2) | P29317 |
| Fas antigen ligand (FasL) | P48023 |
| FAS-associated death domain protein (FADD) | Q13158 |
| Fc receptor-like B (FCRLB) | Q6BAA4 |
| Fibroblast growth factor-binding protein 1 (FGF-BP1) | Q14512 |
| Folate receptor alpha (FR-alpha) | P15328 |
| Folate receptor gamma (FR-gamma) | P41439 |
| Furin (FUR) | P09958 |
| Galectin-1 (Gal-1) | P09382 |
| Glypican-1 (GPC1) | P35052 |
| Granzyme B (GZMB) | P10144 |
| Granzyme H (GZMH) | P20718 |
| Hepatocyte growth factor (HGF) | P14210 |
| ICOS ligand (ICOSLG) | O75144 |
| Insulin-like growth factor 1 receptor (IGF1R) | P08069 |
| Integrin alpha-V (ITGAV) | P06756 |
| Integrin beta-5 (ITGB5) | P18084 |
| Interferon gamma receptor 1 (IFN-gamma-R1) | P15260 |
| Interleukin-6 (IL6) | P05231 |
| Kallikrein-8 (hK8) | O60259 |
| Kallikrein-11 (hK11) | Q9UBX7 |
| Kallikrein-13 (KLK13) | Q9UKR3 |
| Kallikrein-14 (hK14) | Q9P0G3 |
| Ly6/PLAUR domain-containing protein 3 (LYPD3) | O95274 |
| Melanoma-derived growth regulatory protein (MIA) | Q16674 |
| Mesothelin (MSLN) | Q13421 |
| Methionine aminopeptidase 2 (MetAP 2) | P50579 |
| MHC class I polypeptide-related sequence A/B (MIC-A/B) | Q29983,  Q29980 |
| Midkine (MK) | P21741 |
| Mothers against decapentaplegic homolog 5 (MAD homolog 5) | Q99717 |
| Mucin-16 (MUC-16) | Q8WXI7 |
| Nectin-4 (PVRL4) | Q96NY8 |
| Pancreatic prohormone (PPY) | P01298 |
| Podocalyxin (PODXL) | O00592 |
| Pro-epidermal growth factor (EGF) | P01133 |
| Protein CYR61 (CYR61) | O00622 |
| Protein S100-A11 (S100A11) | P31949 |
| Protein S100-A4 (S100A4) | P26447 |
| Proto-oncogene tyrosine-protein kinase receptor Ret (RET) | P07949 |
| Receptor tyrosine-protein kinase erbB-2 (ErbB2/HER2) | P04626 |
| Receptor tyrosine-protein kinase erbB-3 (ErbB3/HER3) | P21860 |
| Receptor tyrosine-protein kinase erbB-4 (ErbB4/HER4) | Q15303 |
| R-spondin-3 (RSPO3) | Q9BXY4 |
| Secretory carrier-associated membrane protein 3 (SCAMP3) | O14828 |
| Seizure 6-like protein (SEZ6L) | Q9BYH1 |
| SPARC (SPARC) | P09486 |
| Stem cell factor (SCF) | P21583 |
| Syndecan-1 (SYND1) | P18827 |
| T-cell leukemia/lymphoma protein 1A (TCL1A) | P56279 |
| TGF-beta receptor type-2 (TGFR-2) | P37173 |
| Tissue factor pathway inhibitor 2 (TFPI-2) | P48307 |
| T-lymphocyte surface antigen Ly-9 (LY9) | Q9HBG7 |
| TNF-related apoptosis-inducing ligand (TRAIL) | P50591 |
| Toll-like receptor 3 (TLR3) | O15455 |
| Transforming growth factor alpha (TGF-alpha) | P01135 |
| Transmembrane glycoprotein NMB (GPNMB) | Q14956 |
| Tumor necrosis factor ligand superfamily member 13 (TNFSF13) | O75888 |
| Tumor necrosis factor receptor superfamily member 4 (TNFRSF4) | P43489 |
| Tumor necrosis factor receptor superfamily member 6B (TNFRSF6B) | O95407 |
| Tumor necrosis factor receptor superfamily member 19 (TNFRSF19) | Q9NS68 |
| Tyrosine-protein kinase ABL1 (ABL1) | P00519 |
| Tyrosine-protein kinase Lyn (LYN) | P07948 |
| WAP four-disulfide core domain protein 2 (WFDC2) | Q14508 |
| Vascular endothelial growth factor A (VEGF-A) | P15692 |
| Vascular endothelial growth factor receptor 2 (VEGFR-2) | P35968 |
| Vascular endothelial growth factor receptor 3 (VEGFR-3) | P35916 |
| VEGF-co regulated chemokine 1 (CXL17) | Q6UXB2 |
| Vimentin (VIM) | P08670 |
| Wnt inhibitory factor 1 (WIF-1) | Q9Y5W5 |
| WNT1-inducible-signaling pathway protein 1 (WISP-1) | O95388 |
| Xaa-Pro aminopeptidase 2 (XPNPEP2) | O43895 |

**Olink® Target 96 Oncology II, Product number: 95700**

Additional file **Table 2.** AUC values for serum and urine supernatant proteins from one-way analysis

| Serum | | Urine | |
| --- | --- | --- | --- |
| Name | AUC | Name | AUC |
| RET | 0.75 | ESM-1 | 0.76 |
| WFDC2 | 0.74 | AREG | 0.72 |
| CXL17 | 0.71 | ERBB2 | 0.66 |
| PPY | 0.70 | S100A11 | 0.63 |
| AREG | 0.70 | CXL17 | 0.62 |
| PVRL4 | 0.70 | FGF-BP1 | 0.62 |
| TFPI-2 | 0.69 | CAIX | 0.62 |
| CD27 | 0.69 | Inc Ctrl 1 | 0.62 |
| TNFSF13 | 0.68 | MK | 0.61 |
| TGFR-2 | 0.68 | MetAP 2 | 0.61 |
| FR-alpha | 0.68 | PPY | 0.61 |
| EPHA2 | 0.67 | SPARC | 0.61 |
| TNFRSF6B | 0.67 | MSLN | 0.60 |
| CD70 | 0.66 | TNFSF13 | 0.60 |
| CTSV | 0.65 | MIA | 0.59 |
| TNFRSF19 | 0.65 | 5'-NT | 0.59 |
| ITGB5 | 0.65 | VEGFA | 0.59 |
| DLL1 | 0.64 | DLL1 | 0.59 |
| WIF-1 | 0.64 | GPNMB | 0.59 |
| MSLN | 0.64 | WISP-1 | 0.58 |
| VEGFR-2 | 0.64 | ERBB4 | 0.58 |
| MK | 0.64 | CRNN | 0.58 |
| IL6 | 0.63 | KLK13 | 0.58 |
| CAIX | 0.63 | CYR61 | 0.57 |
| FR-gamma | 0.62 | IL6 | 0.57 |
| Inc Ctrl 1 | 0.62 | EGF | 0.57 |
| FCRLB | 0.62 | S100A4 | 0.57 |
| RSPO3 | 0.62 | CD160 | 0.57 |
| CD48 | 0.61 | CEACAM1 | 0.56 |
| FASLG | 0.61 | ERBB3 | 0.56 |
| hK11 | 0.61 | TLR3 | 0.56 |
| Gal-1 | 0.61 | MIC-A/B | 0.56 |
| PODXL | 0.61 | LYPD3 | 0.56 |
| ERBB2 | 0.61 | GZMH | 0.55 |
| ERBB3 | 0.60 | CTSV | 0.55 |
| CEACAM5 | 0.60 | CPE | 0.55 |
| TNFRSF4 | 0.60 | hK11 | 0.55 |
| CD160 | 0.59 | TRAIL | 0.55 |
| CYR61 | 0.59 | Gal-1 | 0.55 |
| IFN-gamma-R1 | 0.59 | XPNPEP2 | 0.55 |
| VEGFA | 0.59 | HGF | 0.55 |
| LY9 | 0.59 | TXLNA | 0.55 |
| KLK13 | 0.59 | TGFR-2 | 0.54 |
| CD207 | 0.59 | WIF-1 | 0.54 |
| FADD | 0.58 | WFDC2 | 0.54 |
| GZMH | 0.58 | CXCL13 | 0.54 |
| CEACAM1 | 0.57 | EPHA2 | 0.54 |
| ESM-1 | 0.57 | CD27 | 0.54 |
| SCAMP3 | 0.57 | TFPI-2 | 0.54 |
| ABL1 | 0.57 | TNFRSF19 | 0.54 |
| CPE | 0.57 | Inc Ctrl 2 | 0.53 |
| TRAIL | 0.57 | TNFRSF6B | 0.53 |
| S100A4 | 0.57 | hK8 | 0.53 |
| EGF | 0.56 | LYN | 0.53 |
| LYPD3 | 0.56 | CEACAM5 | 0.53 |
| GZMB | 0.56 | Det Ctrl | 0.53 |
| MAD homolog 5 | 0.56 | GZMB | 0.53 |
| S100A11 | 0.55 | VEGFR-3 | 0.52 |
| TGF-alpha | 0.55 | CDKN1A | 0.52 |
| ITGAV | 0.55 | ABL1 | 0.52 |
| CRNN | 0.55 | FURIN | 0.52 |
| WISP-1 | 0.55 | ITGAV | 0.52 |
| GPNMB | 0.54 | GPC1 | 0.52 |
| MetAP 2 | 0.54 | RSPO3 | 0.52 |
| Det Ctrl | 0.54 | SCAMP3 | 0.52 |
| MIA | 0.54 | TNFRSF4 | 0.52 |
| TCL1A | 0.54 | MUC-16 | 0.52 |
| CDKN1A | 0.54 | IGF1R | 0.51 |
| hK14 | 0.53 | ADAM-TS 15 | 0.51 |
| XPNPEP2 | 0.53 | CD207 | 0.51 |
| Inc Ctrl 2 | 0.53 | ICOSLG | 0.51 |
| MUC-16 | 0.53 | FADD | 0.51 |
| VIM | 0.52 | SYND1 | 0.51 |
| FGF-BP1 | 0.52 | LY9 | 0.51 |
| hK8 | 0.52 | IFN-gamma-R1 | 0.51 |
| SPARC | 0.52 | FR-alpha | 0.51 |
| TLR3 | 0.52 | TGF-alpha | 0.50 |
| ANXA1 | 0.51 | PODXL | 0.50 |
| ERBB4 | 0.51 | VIM | 0.50 |
| ICOSLG | 0.51 | FR-gamma | 0.50 |
| FURIN | 0.51 | VEGFR-2 | 0.50 |
| HGF | 0.51 | Ext Ctrl | 0.50 |
| IGF1R | 0.51 | ADAM 8 | 0.50 |
| ADAM 8 | 0.51 | MAD homolog 5 | 0.50 |
| SCF | 0.51 | FASLG | 0.50 |
| CXCL13 | 0.50 | ANXA1 | 0.49 |
| GPC1 | 0.50 | RET | 0.49 |
| VEGFR-3 | 0.50 | SEZ6L | 0.49 |
| Ext Ctrl | 0.50 | SCF | 0.49 |
| 5'-NT | 0.49 | CD48 | 0.49 |
| LYN | 0.49 | ITGB5 | 0.49 |
| SEZ6L | 0.49 | CD70 | 0.49 |
| MIC-A/B | 0.49 | TCL1A | 0.49 |
| ADAM-TS 15 | 0.49 | hK14 | 0.48 |
| TXLNA | 0.48 | FCRLB | 0.47 |
| SYND1 | 0.47 | PVRL4 | 0.46 |

AUC, Area Under Curve
